# Targeting the arginine metabolic brake enhances immunotherapy for leukaemia

**DOI:** 10.1002/ijc.32028

**Published:** 2019-01-11

**Authors:** Francis Mussai, Rachel Wheat, Evgenia Sarrou, Sarah Booth, Victoria Stavrou, Livingstone Fultang, Tracey Perry, Pamela Kearns, Paul Cheng, Karen Keeshan, Charles Craddock, Carmela De Santo

**Affiliations:** ^1^ Institute of Immunology and Immunotherapy University of Birmingham Birmingham United Kingdom; ^2^ Paul O'Gorman Leukaemia Research Centre, College of Medicine, Veterinary Life Sciences Institute of Cancer Sciences, University of Glasgow United Kingdom; ^3^ Institute of Cancer and Genomic Sciences University of Birmingham Birmingham United Kingdom; ^4^ Bio‐cancer Treatment International Ltd Hong Kong

**Keywords:** arginine, T, CTAG, AML, immunotherapy

## Abstract

Therapeutic approaches which aim to target Acute Myeloid Leukaemia through enhancement of patients’ immune responses have demonstrated limited efficacy to date, despite encouraging preclinical data. Examination of AML patients treated with azacitidine (AZA) and vorinostat (VOR) in a Phase II trial, demonstrated an increase in the expression of Cancer‐Testis Antigens (MAGE, RAGE, LAGE, SSX2 and TRAG3) on blasts and that these can be recognised by circulating antigen‐specific T cells. Although the T cells have the potential to be activated by these unmasked antigens, the low arginine microenvironment created by AML blast Arginase II activity acts a metabolic brake leading to T cell exhaustion. T cells exhibit impaired proliferation, reduced IFN‐γ release and PD‐1 up‐regulation in response to antigen stimulation under low arginine conditions. Inhibition of arginine metabolism enhanced the proliferation and cytotoxicity of anti‐NY‐ESO T cells against AZA/VOR treated AML blasts, and can boost anti‐CD33 Chimeric Antigen Receptor‐T cell cytotoxicity. Therefore, measurement of plasma arginine concentrations in combination with therapeutic targeting of arginase activity in AML blasts could be a key adjunct to immunotherapy.

## Abstract 2

**What's new?**

To date, cellular immunotherapy strategies against acute myeloid leukaemia (AML) have demonstrated limited efficacy. Here, arginine concentrations in AML patients were shown to be significantly lower than in healthy controls, both at diagnosis and during immunomodulatory treatment. Although T cells were able to recognise and be activated by antigens expressed on AML blasts, the low arginine microenvironment acted as a metabolic brake on T cell function and expansion. Targeting arginine metabolism in AML patients should be an adjunct to current treatment strategies in order to enhance host anti‐leukaemia immunity and boost the activity of CAR‐T or adoptive T cell therapies.

## Introduction

T cell immunity plays a key role in the body's defence through the recognition of foreign antigens, expansion in T cells, and signalling through cytokines and cell surface molecules. However, in cancer patients, despite large numbers of abnormal cells being present, immune surveillance is subverted such that T cells fail to recognise the malignant cells or are inhibited in their proliferation or function. A resurgence of cellular immunotherapy strategies has shown the potential for T cells to eradicate leukaemia. Indeed, even relatively crude immune approaches such as allogeneic stem cell transplant in patients with high risk or relapsed AML demonstrate the clinical benefit of T cell activity, despite the risk of Graft *Versus* Host Disease.

Only a few new drugs have been developed for AML that have significantly altered patient outcomes—of these epigenetic modifiers such as azacitidine and vorinostat appear amongst the most promising.^1^, ^2^ One proposed mechanism of action is the upregulation of MHC‐restricted previously undetectable cancer‐testis antigens (CTAG) allowing antigen‐specific T cells to recognise and kill AML blasts.^3^ However, attempts to trigger autologous T cell responses have led to only limited clinical improvements, with the mechanism of failure poorly understood. Furthermore, the mechanisms of how patients’ immune surveillance fails to control leukemic expansion, have not been well characterised.

Recently the interaction between cancer cell metabolism and immunity has become a focus in understanding cancer‐immune evasion. The consumption of amino acids such as arginine have been shown to impact T cell responses in the laboratory setting, yet the translational importance of this mechanism in patients has not been explored.^4^ Here we identify how the failure to address the arginine metabolic microenvironment impacts immune‐modulatory epigenetic therapy or CAR‐T cytotoxicity against leukaemic blasts.

## Materials and Methods

### Patient samples and study approvals

Blood samples were obtained from 80 AML patients ineligible for intensive chemotherapy treated with either azacitidine or azacitidine and vorinostat in a multi‐centre, randomised phase II trial (RAVVA; NCT01617226).^5^ Fresh Peripheral Blood Mononuclear Cells were separated using a Lymphoprep (Alere, Stockport, UK) gradient and stored in liquid nitrogen. Samples from healthy donors were obtained from the University of Birmingham. In accordance with the Declaration of Helsinki, all samples were obtained after written, informed consent prior to inclusion in the study. Regional Ethics Committee (REC Number 10/H0501/39) approval for the study was granted.

### RT‐Q‐PCR analysis

RT‐Q‐PCR was used to detect Cancer‐Testis Antigens and Arginase II in purified patient‐derived AML blasts and cell lines. RNA was extracted using an RNeasy Mini kit (Qiagen, Venlo, Netherlands). cDNA was prepared using SuperScriptTM III Reverse Transcriptase (Invitrogen, Carlsbad, CA) after the manufacturer's instructions. RT‐Q‐PCR was done in duplicate using FAST SYBR Green Master Mix (Applied Biosystems, Foster City, CA) and the Applied Biosystems 7500 Fast Real‐Time PCR system. Analysis of gene expression was calculated according to 2^‐ΔT^ method plotted as arbitrary units of mRNA relative to GAPDH.^13^

**Table:** 

Primer sequences (Eurofins) list		
Human:		
MAGE	5’‐AGTCCTCAGGGAGCCTCC‐3’	Forward
	5’‐ACTCAGCTCCTCCCAGATTT‐3’	Reverse
LAGE	5’‐GCAGGATGGAAGGTGCCC‐3’	Forward
	5’‐CTGGCCACTCGTGCTGGGA‐3’	Reverse
TRAG3	5’‐CCAAAGAGGTTCCCAAGACA‐3’	Forward
	5’‐GCTGTCCCGAAAAGAGACTGG‐3’	Reverse
SSX2	5’‐GTGCTCAAATACCAGAGAAGATC‐3’	Forward
	5’‐TTTTGGGTCCAGATCTCTCGTG‐3’	Reverse
RAGE1	5’‐AGTTCAAACAGGATCAGGAATACCTC‐3’	Forward
	5’‐TGCTCAGTTATCTTCCGCCTTTC‐3’	Reverse
Arginase II	5’‐ATGTCCCTAAGGGGCAGCCTCTCGCGT‐3’	Forward
	5’‐CACAGCTGTAGCCATCTGACACAGCTC‐3’	Reverse
GAPDH	5’‐CCAGCCGAGCCACATCGCTC‐3’	Forward
	5’‐ATGAGCCCCAGCCTTCTC‐3’	Reverse

### IFN‐γ ELISPOT

HLA‐A^*^02+ AML patients were identified by flow cytometry staining (BD Pharmingen, San Jose, CA) of the whole blood. To identify AML patients which contained CTAG responsive T cells IFN‐γ release was measured by ELISPOT (eBioscience, San Diego, CA). In brief, 96‐well PDVF plates were coated with IFN‐γ capture antibody. PBMCs were thawed and plated in the presence of CTAG peptides (20 ng/ml). Influenza GIL and EBV YVL peptides were used as positive controls. After 36 hr, the plates were washed and biotinylated detection antibody added. Avidin‐HRP solution was added and the plate incubated for 45 min after a further wash, substrate solution was added, and left to develop until spots developed, at which point the reaction was terminated by washing, and the plate was left to dry. Spots were counted using a ELISPOT reader.

**Table:** 

Peptides List	
LAGE	SLLMWITQC
RAGE	LKLSGVVRL
TRAG3	ILLRDAGLV
SSX2	KASEKIFYV
NYESO	SLLMWITQC
SART3	LLQAEAPRL
MAGEA10	GLYDGMEHL
MAGEA4	GVYDGREHTV
MAGEA8	KVAELVHFL
MAGEA12	FLWGPRALV
MAGEA1	KVLEYVIKV
MAGEA2	KMVELVHFL
MAGEB1/B2	FLWGPRAYA
MAGEC2	ALKDVEERV

### Statistics

*t*‐Tests (parametric) were used to determine the statistical significance of the difference in paired observations between two groups (GraphPad Prism, San Diego, CA). *p* values are two‐tailed and where values were <0.05,they were considered statistically significant. A Kaplan–Meier survival curve was generated using GraphPad PRISM.

Methodological information regarding cell lines, flow cytometric analysis, mixed lymphocyte reactions, our AML murine models, ELISA and engineered T cells can be found in the supplements.

## Results

### T cells are functionally exhausted despite enhanced cancer‐testis antigen expression on AML blasts

Epigenetic modifying drugs such as azacitidine and vorinostat may boost the anti‐leukaemia T cell immune response by upregualting Cancer‐Testis Antigens expression.^6^, ^7^ First we showed that treatment of AML cell lines with Azacitidine and Vorinostat can upregulate MAGE, LAGE, RAGE, TRAG3, and SSX2 CTAGs on AML tumour cell lines (Supporting Information Fig. S1 *a*). Recently we investigated the clinical efficacy of combining azacitidine with vorinostat in a Phase II trial in AML and high risk MDS patients.^5^ Although a number of clinical responses were seen, vorinostat provided no additional clinical benefit in terms of overall survival. We hypothesised that these drugs should have upregulated CTAGs in AML blasts making them a more attractive immune target. However, the mechanism of why the T cell response is inadequate to control the disease is unknown.

**Figure 1:**
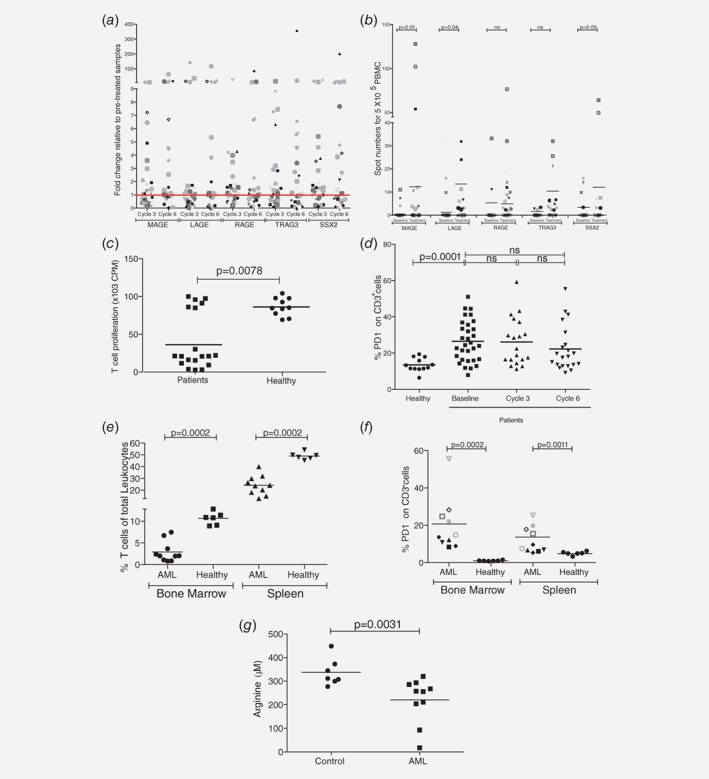
Azacitidine and Vorinostat induce an upregulation of Cancer‐Testis Antigens in AML blasts. (*a*) AML patients treated with azacitidine and vorinostat have increased expression of MAGE, LAGE, RAGE, TRAG3 and SSX2 Cancer‐Testis Antigens in AML blasts at Cycle 3 and Cycle 6 compared to at the time of study enrolment. Expression assessed by qRT‐PCR in *n* = 40 patients. Baseline expression is shown by the red line (fold change 1). (*b*) Antigen‐specific T cells from patients treated with azacitidine and vorinostat demonstrated increased IFN‐γ release in response to CTAG peptide stimulation *ex vivo*. IFN‐γ positive T cells were measured by ELISPOT. (*c*) T cells from AML patients have a reduced proliferative capacity compared to those from healthy donors, in response to CD3/CD28 antibody stimulation. (*d*) Expression of exhaustion marker PD1 was assessed on CD3+ T cells from the blood of AML patients during treatment. (*e*) MLL‐AF9 AML engrafted mice have significantly reduced T cell numbers in the bone marrow and spleen compared to healthy controls as assessed by flow cytometry. (*f*) T cells from the bone marrow and spleens of engrafted mice have increased PD1 expression compared to healthy controls, as assessed by flow cytometry. (*g*) AML mice have a significant reduction in plasma arginine concentrations compared to healthy controls.

CTAGs are expressed in the context of HLA‐A*02. Analysis of AML blasts from HLA‐A*02 patients on the AZA/VOR clinical trial after three and six cycles of therapy identifies upregulation of the five families of CTA expression (MAGE, RAGE, LAGE, SSX2 and TRAG3) compared to before drug administration in individual patients (Fig. 1 *a*) confirming our hypothesis. On recognition of target antigens, classically T cells respond through cell division, IFN‐γ release and cytotoxicity. Patient‐derived PBMC from 30 HLA‐A*02 patients were isolated before and during treatment (Supporting Information Fig. S1 *b*), and pulsed with relevant CTAG peptides *ex vivo*. Increased activated anti‐CTAG T cells for MAGE, LAGE, and SSX2 antigens were particularly prominent as assessed by release of IFN‐γ release after AZA/VOR (Fig. 1 *b*, Supporting Information Fig. S1 *c* and *d*). However, although the drugs led to significant increase in antigen‐specific T cell activation compared to baseline, surprisingly, these responses are not greater than those of healthy donors (Supporting Information Fig. S2 *a*).^8^

We hypothesised that T cells in patients may be functionally exhausted—a state characterised by a failure to expand in response to stimulation and upregulation of cell surface inhibitory receptors such as PD1, LAG3 and TIM3.^16^ First, we identified that in the majority of patients T cells had a significantly reduced ability to proliferate (*p* = 0.0078) compared to those from healthy donors (Fig. 1 *c*). Phenotyping demonstrated upregulated PD1 expression (*p* = 0.0001) throughout treatment, with no changes in LAG3 or TIM3 (Fig. 1 *d*, Supporting Information Fig. S2 *b* and *c*). Thus, the expected physiological expansion of activated T cells in response to antigens on AML blasts is absent.

### The failure to address the low arginine microenvironment impairs anti‐CTAG clinical responses

Arginine metabolism is a pathway which is aberrant in AML blasts and can influence the immune microenvironment.^4^, ^9^ Using an immunocompetent, syngeneic model of AML, we showed that significant numbers of AML blasts (CD45.2+) were detectable in the bone marrow (median 80%) and spleens (median 40%) of mice, analogous to presentation of AML in patients (Supporting Information Fig. S2 *d* and *e*). AML bearing mice showed significant reductions in the percentage of T cells in the bone marrow (*p* = 0.0002) and spleens (*p* = 0.0002; Fig. 1 *e*) which also expressed increased PD1 (bone marrow *p* = 0.0002, spleens *p* = 0.0011) consistent with our human findings (Fig. 1 *f*). The mice had significant reductions in plasma arginine compared to controls (*p* = 0.0031; Fig. 1 *g*).

**Figure 2:**
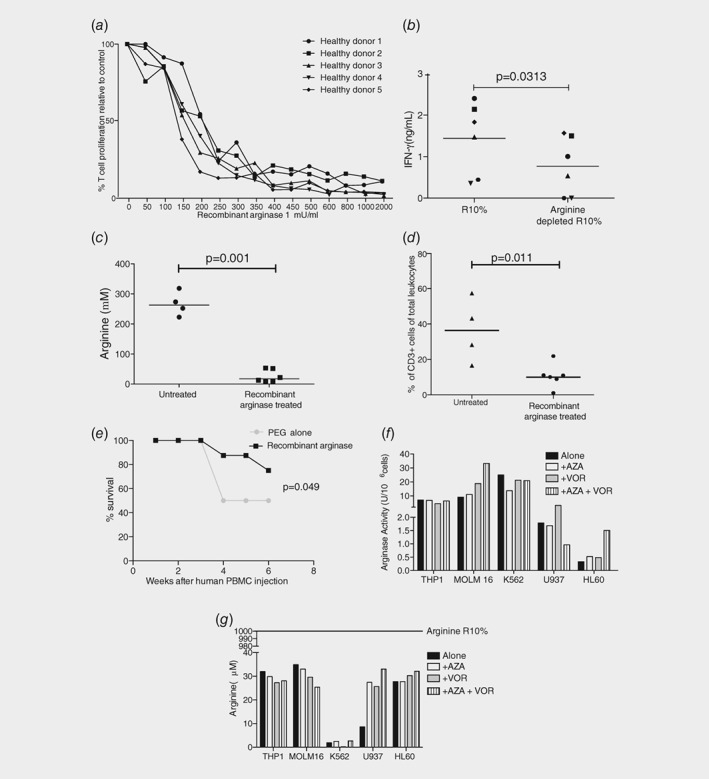
Arginase activity inhibits allogenic T cell responses. (*a*) Recombinant arginase leads to inhibition of T cell proliferation in allogeneic mixed leukocyte reactions. Five representative donors shown. (*b*) Culture of T cells from healthy donors in complete media (R10%) or arginine depleted media leads to a significant reduction in IFN‐γ release in response to CD3/CD28 antibody stimulation. (*c*) Administration of recombinant arginase to NOG‐SCID mice engrafted with human lymphocytes leads to a significant reduction in plasma arginine. (*d*) Recombinant arginase leads to a significant reduction in the frequency of T cells in the spleens of NOG‐SCID mice engrafted with human lymphocytes, as assessed by flow cytometry. (*e*) Kaplan–Meier survival curves showing NOG‐SCID mice engrafted with human T cells have increased survival after treatment with recombinant arginase. (*f*) Arginase activity of AML cell lines after treatment with azacitidine and/or vorinostat. (*g*) Concentration of arginine in the supernatants of AML cell lines after treatment with azacitidine and/or vorinostat.

Nonself‐antigen presentation by an allogeneic MHC is a profound model of T cell stimulation and proliferation. To investigate the specific impact of plasma arginine concentrations, we first identified *in vitro* that T cell expansion in response to allogeneic dendritic cells was profoundly inhibited by the addition of recombinant human arginase to mimic AML blast activity (Fig. 2 *a*) with a corresponding reduction in activation‐induced IFN‐γ release (Fig. 2 *b*). Next equal numbers of human T cells (10 × 10^6^) were engrafted into NOG‐SCID mice. T cells rapidly engrafted in the spleen (*n* = 14 days). Analogous to the arginine deplete microenvironment found in patients at diagnosis, administration of recombinant arginase led to a significant reduction in serum arginine (*p* = 0.001; Fig. 2 *c*) and a failure of T cells to expand (*p* = 0.011; Fig. 2 *d*). Notably overall survival was prolonged by the effects of arginine deprivation on T cells in these mice which otherwise develop Graft *Versus* Host Disease lethality from T cell responses to allo‐antigens (*p* = 0.049; Fig. 2 *e*).

**Figure 3:**
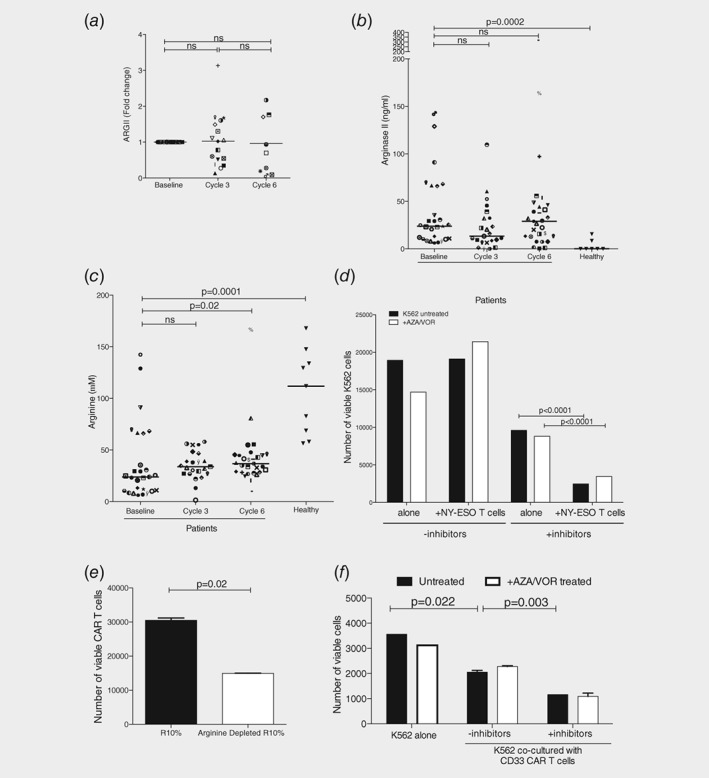
Inhibition of arginine metabolism enhances T cell immunotherapy cytotoxicity against AML. (*a*) AML patients treated with azacitidine and vorinostat have no significant changes in Arginase II expression in AML blasts at Cycle 3 and Cycle 6 compared to at the time of study enrolment. Expression assessed by qRT‐PCR. (*b*) Plasma Arginase II concentrations are elevated in AML patients, compared to healthy controls, and are not significantly altered after cycles of azacitidine and vorinostat treatment. (*c*) Plasma arginine concentrations are decreased in AML patients, compared to healthy controls, and are not significantly altered after cycles of azacitidine and vorinostat treatment. (*d*) Anti‐NY‐ESO antigen‐specific T cells were cultured with K562 (target) AML cells. The numbers of viable K562 cells was significantly reduced if antigen‐specific T cells are used in combination with inhibitors of AML arginine metabolism (+inhibitors: L‐NMMA and NOHA). Pretreatment with azacitidine and vorinostat did not affect cytotoxicity. (*e*) Anti‐CD33 CAR‐T cell proliferation is inhibited when cultured in arginine depleted media. CAR‐T cell numbers were counted by flow cytometry after 72 hr. (*f*) Anti‐CD33 CAR‐T cells were cultured with K562 (target) AML cells. The numbers of viable K562 cells were reduced by CAR‐T cells alone. The numbers of viable K562 cells was further reduced if AML arginine metabolism is inhibited (+inhibitors: L‐NMMA and NOHA). Pretreatment with azacitidine and vorinostat did not affect cytotoxicity.

Next we investigated the contribution of arginine metabolism on T cell responses in the trial patients. Arginase II can be regulated by histone deacetylases in nonmalignant cells, therefore we hypothesised that the AZA/VOR may also downregulate arginase activity in AML blasts.^10^ Culture of AML cell lines with azacitidine and vorinostat did not decrease arginase enzyme activity (Fig. 2 *f*) or supernatant arginine concentrations (Fig. 2 *g*). Consistent with this, analysis of patients’ AML blasts demonstrated no change in Arginase II over time (Fig. 3 *a*). Patients maintained an increase in plasma Arginine II enzyme (Fig. 3 *b*, *p* = 0.0002) and significant reduction in arginine concentrations (Fig. 3 *c*, *p* = 0.0001) throughout the trial. Therefore, the arginine deplete environment remains throughout therapy and can inhibit antigen‐dependent T cell responses *in vitro* and *in vivo*.

### Targeting arginine metabolism enhances T cell immunotherapy responses

Arginine catabolism appears to be a critical factor in holding back the immune response to antigen on AML blasts. We therefore investigated if targeting Arginase II enzyme activity could enhance the T cell response *in vitro*. L‐NMMA and NOHA are two arginine analogues which can lead to reversible inhibition of arginine metabolism *in vitro*. Human T cells and purified AML blasts were first co‐cultured, in the presence of stimulatory allogeneic dendritic cells. AML blasts inhibited T cell proliferation replicating our murine findings. However, inhibition of arginase activity with L‐NMMA and L‐NOHA led to restoration of T cell proliferation. (Supporting Information Fig. S3 *a*).

NY‐ESO‐1 is an established AML‐associated cancer‐testis antigen that can be presented by HLA‐A2 positive blasts (Supporting Information Fig. S3 *b*).^6^ Patient antigen‐specific CTAG responses can be modelled through the selection and expansion of anti‐NY‐ESO T cells *ex vivo*, a process latterly used to generate adoptive immunotherapy. In co‐cultures engineered anti‐NY‐ESO T cells alone had no effect on K562 AML viability, including after K562 cells were pretreated with azacitidine and vorinostat (Fig. 3 *d*). Although no additional upregulation of NY‐ESO was detected on K562 (Supporting Information Fig. S3C), the arginase inhibitors enhanced engineered anti‐NY‐ESO T cell cytotoxicity, leading to a further significant reduction in the number of viable K562 cells (*p* = 0.001; Fig. 3 *d*).

We hypothesised that arginine metabolism may be similarly contributing to the failure of other antigen‐specific T cell immune therapies. Clinical responses with CAR‐T cell therapy for AML have lagged behind the notable successes in Acute Lymphoblastic Leukaemia, despite the presence of viable antigenic targets.^11^, ^12^ The mechanism of failure is poorly understood. CAR‐T cell proliferation was assayed in normal and low arginine conditions. Low arginine conditions significantly inhibited CAR‐T cell proliferation (Fig. 3 *e*). Incubation of anti‐CD33 CAR‐T cells with K562 led to a reduction in the number of viable AML cells (*p* = 0.022; Fig. 3 *f*). Inhibition of arginine metabolism enhanced CAR‐T cell cytotoxicity against the AML cells and led to a further significant reduction in viable AML cells (*p* = 0.003; Fig. 3 *f*) Azacitidine and vorinostat did not affect CAR‐T cell cytotoxicity. Therefore, like autologous immune responses, engineered T cells have the capacity to recognise and kill AML blasts but their full potential is not realised until arginine metabolism is targeted and inhibited.

## Discussion

The aberrant expression of Cancer‐Testis Antigens offers the potential to use a patient's immune system, or engineered immune therapies, to target AML. A number of CTAs including SCP1, MAGE, SPAN‐Xb, SLLP1 and PRAME CTAs have been identified in unmanipulated AML blasts and may correlate with an improved overall survival.^13^, ^14^, ^15^ In the RAAVA Phase II trial patients, we showed that azacitidine and vorinostat increased expression of a number of CTAGs not previously reported such as LAGE, TRAG3 and SART3. Circulating T cells demonstrated the capacity to be activated in response to target antigen, thus epigenetic modifiers can act as immune modulators. However, as in previous clinical studies, two observations are pertinent which have not been adequately justified to date. The first is that the number of CTAG responding T cells is small despite the large blast and antigenic burden. In the case of MAGE, these antigen‐specific T cells may constitute less than 1% of the total CD8+ T cell pool, an insignificant number considering the rapid rate of leukaemia cell division. The second observation is that despite the presence of antigen and circulating responsive T cells there is no correlation with overall response to epigenetic modifying drugs. For adoptive T cell combinations, epigenetic modulation has mostly been tested in solid tumours, with only one phase I trial reporting encouraging results using decitabine to boost a NY‐ESO‐1 vaccine for human ovarian cancer.^16^, ^17^, ^18^

Our findings are the first to report the clinical impact of the low arginine microenvironment on immune therapy approaches in patients, and extend our preclinical findings on the role of tumour arginine metabolism in suppressing immunity.^4^, ^19^ In the case of AML blasts, arginine catabolism is a function of their Arginase II enzyme,^9^ in combination with a failure to express a full complement of arginine recycling enzymes, making blasts reliant on extracellular sources of arginine.^20^ The increased metabolic requirement for arginine by activated T cells is clear, with arginine also contributing to T cell survival through BAZ1B, PSIP1 and TSN signalling.^21^ In our study, no correlation of T cell responses and patient clinical outcome was seen, suggesting that any immune responses are inadequate in the low arginine microenvironment. Attempts to reactivate autologous immunity or enhance cellular immunotherapy therefore require not only availability of antigen expression but must also address the immunosuppressive metabolic microenvironment which acts as constraint to cellular expansion. Although epigenetic modifying drugs may influence multiple pathways, the study demonstrated they cannot help to downregulate Arginase II expression.

Our study has important translational implications and help to explain the functional impairment in T cells seen in trials of peptide vaccines in AML patients or more recently with CAR‐T cells.^22^ A more direct approach to metabolic modulating therapy would be to target the Arginase II enzyme's active site. A multitude of novel inhibitors have sought to target arginase enzymes, mostly with limitations such as reversibility, a failure to cross the cell membrane, or the need to block both Arginase and INOS enzymes if present in cancer cells.^23^, ^24^ The most clinically advanced arginase inhibitor is CB‐1158 (Calithera), an orally active agent that is undergoing clinical trial alongside checkpoint inhibitors in patients with advanced solid tumours (NCT02903914). Alternatives could include screening of patients’ plasma for arginine concentrations to ensure T cell therapies are administered at an optimal time point, or administration of T cell therapies with an arginine supplemented diet. The later approach has been used with mixed results to enhance healing postsurgery, but may have untoward effects of feeding tumour metabolism. As a number of cancers which are targets for T cell immunotherapies deplete arginine through aberrant arginine recycling pathways, future strategies should seek to urgently address this significant metabolic challenge.^25^

## Author contributions

F.M. and C.D.S designed the study, performed research, analysed data and wrote the study. R.W., S.B., L.F., E.S., V.S., T.P., K.K. performed research. C.C. provided access to patient samples as Chief Investigator of the RAVVA trial. F.M., C.C., K.K., and P.K. secured ethical approval for the study.

## Supporting information


**Supplementary Figure 1** (*a*) Upregulation of CTAG expression in AML cell lines after AZA/VOR treatment as assessed by qPCR. Baseline expression illustrated with red line. (*b*) Example flow cytometry gating for HLA‐A*02 identification on AML blasts from patients. (*c*) NY‐ESO and SART3 antigen‐specific T cells from patients treated with azacitidine and vorinostat demonstrated increased IFN‐γ release in response to peptide stimulation *ex vivo*. IFN‐γ positive T cells were measured by ELISPOT. (*d*) MAGE‐specific T cells from patients treated with azacitidine and vorinostat demonstrated increased IFN‐γ release in response to CTAG peptide stimulation *ex vivo*. IFN‐γ positive T cells were measured by ELISPOT.

**Supplementary Figure 2** (*a*) Antigen‐specific T cells from patients treated with azacitidine and vorinostat have comparable IFN‐γ release in response to CTAG peptide stimulation *ex vivo* compared to healthy donors. IFN‐γ positive T cells were measured by ELISPOT. (*b*) LAG3 and TIM3 (*c*) were assessed on CD3+ T cells from the blood of AML patients during treatment. (*d*) Flow cytometric gating strategy for the detection of CD45.2(AML blasts) and CD45.1 (CD3+ T cells) in the spleens of MLL‐AF9 syngeneic AML murine model. (*e*) Percentage of AML blasts in the bone marrow and spleens of MLL‐AF9 syngeneic murine model.

**Supplementary Figure 3** (*a*) T cell proliferation is suppressed when co‐cultured with AML blasts from patients. Inhibition of arginine catabolism with LNMMA and NOHA rescues T cell proliferation in the allogeneic mixed leukocyte reactions. 7 representative patients shown. (*b*) Expression of HLA‐A2 in K562 cells before and after treatment with AZA/VOR. (*c*) Example flow cytometry gating for HLA‐A*02 identification on K562 after treatment with azacitidine and vorinostat.

**Appendix S1:** Supporting information
