# Supplementary material for: Targeting the arginine metabolic brake enhances immunotherapy for leukaemia
Source: Int J Cancer. 2019 Jan 11;145(8):2201–8. doi: 10.1002/ijc.32028 (PMC6767531; doi:10.1002/ijc.32028)
Supplement: Supplementary file 4 — Appendix S1: Supporting information [file IJC-145-2201-s001.docx]

**Supplementary Methods:**

*Cell lines*

Cell lines THP-1, U937, MOLM16, K562, HL60 were obtained from DSMZ (Germany). Cells were cultured in RPMI-1640 (Invitrogen, CA, USA) with 10% heat-inactivated fetal bovine serum, glutamine (1x), sodium pyruvate (1x) and Penicillin-Streptomycin (RPMI 10%) using T-75 flasks kept in a humidified air atmosphere with 5% CO_2_ at 37^o^C. Where indicated AML cell lines were cultured with azacitidine (500nM) for 72hours and then vorinostat (1mM) for 24hours, before harvesting, and washing before replating with T cells, where indicated.

*Flow cytometric analysis*

PBMCs were stained with anti-human CD3, CD4, CD8, CD33, CD34, PD1, LAG3, TIM-3, CD45.1 and CD45.2 antibodies (BioLegend) on ice for 30 minutes, as indicated. Propidium iodide (PI) was used to assess viability. Intracellular staining for phosphorylated STAT proteins was determined according to manufacturer’s instructions (BD Phosflow T cell Activation Kit). Cells were analysed using a Cyan-ADP flow cytometer (Beckman-Coulter) and analysed using FlowJo software (Tree Star Inc).

*Mixed Lymphocyte Reaction*

Dendritic cells were derived by initial isolation of peripheral blood mononuclear cells from healthy donor blood using Lymphoprep density gradient centrifugation, followed by sorting with CD14+ MACS bead sorting (Miltenyi Biotech). Sorted cells were confirmed as >99% pure by flow cytometry. Dendritic cells were generated by culturing monocytes in complete media with the addition of 50ng/ml GM-CSF (Peprotech) and 1000U/ml Il-4 for 5 days in 6 well plates. Sorted T lymphocytes (2x10^5^) were then cultured with allogeneic irradiated (5000 rad) dendritic cells, in complete media in 96 well flat bottom plates. The suppressive ability of MACS sorted patients’ blasts was assessed by co-culturing purified cells together with the T cells. N_G_-hydroxy-L-arginine (0.5mM, NOHA, Cayman USA) and L-N_G_-monomethyl arginine (0.5mM, L-NMMA, Cayman USA) were added where indicated to inhibit arginine metabolism. 0.5mM NOHA and L-NMMA has been established as dose with no effect on AML or T cell viability. Cells were incubated at 37^o^C, 5% CO_2_ for 4 days and then 1μCi/ well ^3^H-thymidine (Perkin Elmer Life Sciences) was added for 15-18 hours. ^3^H-thymidine incorporation was measured using a TopCount reader (Perkin Elmer). The suppressive ability of arginase was assessed by the addition of 0-2000ng/ml pegylated recombinant arginase (BCT-100, Bio-Cancer Treatment International, Hong Kong) at the start of cultures. Data are expressed as a percentage of T cell proliferation driven by allogeneic irradiated DCs in the presence of arginase, compared with allo-reactive T cell proliferation in the absence of arginase (100%).

*AML syngeneic murine model*

MLL-AF9 cells were cultured in RPMI with 20% fetal bovine serum and 20% WEHI-conditioned medium. (11, 12) 1x10^6^ MLL-AF9 cells were transplanted into sublethally irradiated (4.5Gy) B6.SJL-Ptprc^a^ Pepc^b^/BoyJ (CD45.1+) mice recipients and sacrificed at day 17 post bone marrow transplant. AML donor cells (CD45.2+) and T cell frequency were identified by flow cytometry of sacrificed mice. The University of Glasgow Animal Welfare and Ethical Review Board (AWERB) approved all animal protocols in this study. Procedures were carried out in accordance with UK Home Office Guidelines.

*Allogeneic T cell proliferation murine model*

NOD/Shi-scid/IL-2R SCIDγnull (NOG) mice (Biomedical Services Unit, University of Birmingham) aged 10-14 weeks were irradiated with 1.25 Gy. One day later 10x10^6^ peripheral blood lymphocytes from healthy human donors were injected into the tail vein. 5mg/kg BCT-100 was injected intraperitoneally (i.p.) 3x weekly. Spleens were harvested following sacrifice after 14 days of treatment, disrupted mechanically, and red cells lysed using Red Cell Lysis Buffer (Qiagen). T cell engraftment was defined by the detection of human CD3+ cells using flow cytometry. The Birmingham Biomedical Ethics Review Subcommittee (BERSC) approved all animal protocols in this study. Procedures were carried out in accordance with UK Home Office Guidelines.

*ELISA*

The concentration of arginine in human and murine plasma was quantified using a competitive enzyme linked immunoassay (K7733, Immunodiagnostik, Germany) according to the manufacturers’ instructions. In brief, the assay uses a competitive enzyme immunoassay in which L-arginine is derivatized from samples, and competes with an L-arginine-tracer for binding of polyclonal antibodies, in the microtiter wells. The concentration of the tracer-bound antibody is inversely proportional to the L-arginine concentration in the samples. Arginase II concentrations in human plasma was measured using a sandwich ELISA (AntibodiesOnline, Atlanta, GA) according to the manufacturer’s instructions. The concentration of IFN-γ (BioLegend) was similarly measured by ELISA according to the manufacturer’s instructions.

*NY-ESO-1 specific TCR engineered T cell generation*

Human CD3^+^ T cells selected from PBMC of a healthy donor were gene modified with a pMP71 retroviral construct encoding an HLA-A*0201-restricted TCR specific for the NY-ESO-1 peptide epitope SLLMWITQC as described previously.(14)Transduction efficiency was determined at 72 h using an antibody against mouse TCRβ chain (BD Biosciences; H57-597; 1:100). NY-ESO-1 T cells were stimulated with T2 cells pulsed with NY-ESO-1 peptide (90 minutes at 37 °C) in the presence of K562 cell pre-treated with azacitidine/vorinostat, and with NOHA and L-NMMA. K562 viability was assessed after 4 days by flow cytometry staining.

*Chimeric Antigen Receptor T cell generation*

Phoenix Ampho cells (retroviral packaging cell line) were cultured in DMEM with 10FCS and 1% L-glutamine. After trypsinisation Ampho cells were re-plated and transfected with retroviral CAR plasmid expressing a 4-1BB second generation Chimeric Antigen Receptor targeting CD33 in combination with a truncated CD34 for identification and isolation of CAR expressing cells. PBMC from healthy donors were obtained by density gradient separation of blood samples and activated with CD3/CD28 Dynabeads® (Life Technologies) and 100u/ml IL-2 (Peprotech). Transfected Phoenix Ampho cell line supernatants were collected and used to transduce activated T cells in the presence of Retronectin (Takara). Following expansion for 4 days, CAR-T cell purity was checked by flow cytometry gating on the truncated CD34 positive population. CAR-T cells were flow sorted into pure populations using a CD34 MACS beads (Miltenyi) and plated within target K562 cells pre-treated with azacitidine/vorinostat, and with NOHA and L-NMMA. K562 viability was assessed after 4 days by flow cytometry staining.

*Arginase II activity*

The activity of arginase II present within AML blasts, was determined by measuring the conversion of arginine into urea. Patient AML blasts were cultured in RPMI+10% FCS at 37^o^C, 5% CO_2_. After 24 hours the supernatants were collected and the cells pelleted and lysed with 50 μl of buffer containing 0.1% Triton X-100, 5 μg pepstatin, 5 μg aprotinin and 5 μg antipaina. The same buffer was added to 50 μl of cell supernatants or patient plasma. The samples were placed on a 37^o^C heat block for 30 minutes before centrifugation at 14,000 rpm and collection of the supernatants. To activate the arginase enzyme, buffer containing Tris-HCl (25 mM) and MnCl_2_ (10 mM) was added and heated to 56^o^C for 10 minutes. L-arginine (0.5M, Sigma) was added and the samples were heated for 1 hour at 37^o^C. The hydrolysis of arginine was stopped with 800 μl of an acid solution mixture (H_2_SO_4_:H_3_PO_4_:H_2_O, 1:3:7). The amount of urea produced was determined using 9% α-isonitrosopropriophenone and compared to a standard curve with absorbance measured at 540 nm
